# NATURAL RESOURCES: Reversing Human Impacts on Fish Evolution

**DOI:** 10.1289/ehp.117-a197

**Published:** 2009-05

**Authors:** John Tibbetts

Next time you catch a “big one,” throw it back. This practice might seem counterintuitive, given that minimum size regulations favor keeping the larger fish, but it could reap long-range dividends for fish and humans alike, according to a study published online 4 March 2009 ahead of print in the *Proceedings of the Royal Society B*. Since 1990, scientists have observed that fish are getting smaller and growing more slowly as humans have continued to harvest the largest fish in wild stocks. Selecting out the large fish from a population sets the stage for earlier adult maturation, which means smaller fish are producing fewer eggs and offspring. This, in turn, could shrink many wild harvests. But the news is not all bad: The new study by David O. Conover, dean and director of the School of Marine and Atmospheric Sciences at Stony Brook University in New York, indicates fishery-induced genetic change can be slowly reversed.

Conover and his colleagues report on an empirical simulation experiment wherein captive populations of Atlantic silverside (*Menidia menidia*), a bait fish, were caught off the coast of the Great South Bay, New York. Groups of fish were selectively culled for the largest fish, mimicking the practice of most fisheries. The populations evolved smaller body size during the first five generations, and the smaller fish became less fertile because they produced fewer eggs.

“In our experiment,” says Conover, “the females were getting so small and producing eggs that were so small that the survival of those eggs and larvae were reduced dramatically. We would have eventually driven our own study population into extinction if we hadn’t stopped the large harvesting after five generations. We were struggling to produce enough fish for the sixth generation.”

From the sixth through tenth (and final) generation, fish were harvested at random. The fish populations showed a slow but significant increase in size, although they did not reach full recovery. The researchers estimate that it would take about 12 generations of random harvests for the body size of this fish to return to normal. Harvested fish species typically have generation times of 3–7 years, so recovery of some overfished populations could take 3–8 decades, given a size decline of the magnitude induced in this study.

Chris Darimont, a postdoctoral fellow at the University of California, Santa Cruz, says, “Scientists have almost exclusively studied how exploited populations have undergone undesirable changes such as smaller sizes or less productive breeding schedules. But this study looks at what happens when a population is released from size-selective predation.” Darimont wrote that human predation is rapidly accelerating the rate of observable trait changes in commercially harvested species in the 20 January 2009 issue of the *Proceedings of the National Academy of Sciences*.

Fish provides more than 2.9 billion people with at least 15% of their average per-capita animal protein intake, according to the *World Fisheries and Aquaculture 2008* report by the Food and Agriculture Organization of the United Nations. The report says that 80% of all marine fish stocks for which assessment information is available are fully exploited or overexploited.

To help recover an overexploited population, fishery managers could establish “slot limits” that allow harvesting only of mid-size fish, according to Conover. Individual fish above a maximum size or below a minimum size would have to be returned. Slot limits apply evolutionary pressure on young fish to grow more quickly so they become larger and mature earlier. “The individual fish that survive the window would be allowed to grow large, and fecundity goes up exponentially with length,” says Conover. Maine’s lobster fishery has a slot limit.

Slot limits for some fisheries could have direct human health benefits, Conover says. In some fish such as tuna, the largest and oldest accumulate the highest levels of pollutants such as mercury. By not commercially harvesting the largest and most contaminated fish, people would be less likely to eat them.

